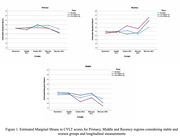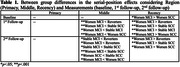# Serial‐position effect in participants cognitively unimpaired with Subjective Cognitive Complaints (SCCs) and with Mild Cognitive Impairment (MCI) that revert, remain stable or worsen: Evidence from the Compostela Aging study (CompAS)

**DOI:** 10.1002/alz.091559

**Published:** 2025-01-03

**Authors:** Arturo X. Pereiro Rozas, Maria Campos‐Magdaleno, Cristina Lojo‐Seoane, Lucía Pérez‐Blanco, Sabela Carme Mallo, Sonali Arora, Fátima Fernández‐Feijoo, David Facal

**Affiliations:** ^1^ Applied Cognitive Neuroscience and Psychogerontology group, Health Research Institute of Santiago de Compostela (IDIS), Santiago de Compostela Spain; ^2^ Deparment of Developmental and Educational Psychology, University of Santiago de Compostela, Santiago de Compostela Spain; ^3^ Department of Developmental and Educational Psychology, University of Santiago de Compostela, Santiago de Compostela Spain

## Abstract

**Background:**

Higher recency effect in episodic memory (EM) has been found for MCI and dementia (Martin et al., 2013; Campos et al., 2016). Additional research should be conducted on the pre‐symptomatic stages and on their ability to predict progression to cognitive decline. Our aim is to analyze the differences in the serial‐position effect in cognitively unimpaired and MCI participants that revert, remain stable or worsen.

**Method:**

A sample of 162 participants from the CompAS were classified in five groups considering change or stability from its baseline diagnoses to the 2^nd^ follow‐up (48‐76 months after baseline) as follows: Stable‐SCC (SCC who remain stable; N = 97), Worsen‐SCC (SCC who worsening to MCI/dementia; N = 32), Stable‐MCI (MCI who remain stable; N = 13), Worsen‐MCI (MCI who worsening to dementia; N = 8), and Reverters (participants who revert from MCI; N = 12). The Spanish version of the CVLT were administered and corrected regional scores were computed (see Campos et al., 2016). A mixed model was carried out to test group differences considering the intrasubject factors Regions (Primacy, Middle, Recency) and Measurements (baseline, 1^st^ follow‐up, 2^nd^ follow‐up).

**Results:**

Significant effects was found for the interaction between Group*Region*Measurement also achieved significance (*F*(16,628) = 1.87; *p*<.021; *η_p_^2^* = .045, *observed power* = .957). Differences between Measurements were not found in any Region for Reverters and Stable‐SCC. In both, Stable‐MCI and Worsen‐SCC groups differences showed better performance in 1^st^ than in 2^nd^ follow‐up in Middle and poor retrieval in Baseline than in 2^nd^ follow‐up in Recency regions. Worsen‐MCI showed better retrieval in Baseline than in 2^nd^ follow‐up for the Middle region but poor retrieval in Baseline than in 1^st^ and 2^nd^ follow‐up for the Recency region (see Figure 1). Group differences were found mainly in Middle and Recency regions (see Table 1) and in the 1^st^ and particularly 2^nd^ follow‐up showing poor retrieval particularly for the Worsen‐MCI.

**Conclusions:**

Recency effect becomes prominent compensatory mechanism as cognitive decline progress. Retrieval in Middle region decrease only MCI participants. Poor retrieval in Middle region scores could be useful to improve identification of SCC at risk.